# Biochar affects compressive strength of Portland cement composites: a meta-analysis

**DOI:** 10.1007/s42773-024-00309-2

**Published:** 2024-03-06

**Authors:** Zhihao Zhao, Ali El-Naggar, Johnson Kau, Chris Olson, Douglas Tomlinson, Scott X. Chang

**Affiliations:** 1 Department of Renewable Resources, University of Alberta, 442 Earth Sciences Building, Edmonton, AB T6G 2E3 Canada; 2 Department of Soil Sciences, Faculty of Agriculture, Ain Shams University, Cairo, 11241 Egypt; 3 State Key Laboratory of Subtropical Silviculture, Zhejiang A&F University, Hangzhou, Zhejiang, 311300 China; 4 Department of Civil Engineering, University of Alberta, 6-255 Donadeo Innovation Centre For Engineering, Edmonton Alberta, T6G 2H5 Canada; 5 Innovative Reduction Strategies Inc, Northtown PO, PO Box 71022, Edmonton Alberta, AB T5E 6J8 Canada

**Keywords:** Biochar, Portland cement, Cement, Constructure, Compressive strength

## Abstract

One strategy to reduce CO_2_ emissions from cement production is to reduce the amount of Portland cement produced by replacing it with supplementary cementitious materials (SCMs). Biochar is a potential SCM that is an eco-friendly and stable porous pyrolytic material. However, the effects of biochar addition on the performances of Portland cement composites are not fully understood. This meta-analysis investigated the impact of biochar addition on the 7- and 28-day compressive strength of Portland cement composites based on 606 paired observations. Biochar feedstock type, pyrolysis conditions, pre-treatments and modifications, biochar dosage, and curing type all influenced the compressive strength of Portland cement composites. Biochars obtained from plant-based feedstocks (except rice and hardwood) improved the 28-day compressive strength of Portland cement composites by 3–13%. Biochars produced at pyrolysis temperatures higher than 450 °C, with a heating rate of around 10 C min^-1^, increased the 28-day compressive strength more effectively. Furthermore, the addition of biochar with small particle sizes increased the compressive strength of Portland cement composites by 2–7% compared to those without biochar addition. Biochar dosage of < 2.5% of the binder weight enhanced both compressive strengths, and common curing methods maintained the effect of biochar addition. However, when mixing the cement, adding fine and coarse aggregates such as sand and gravel affects the concrete and mortar's compressive strength, diminishing the effect of biochar addition and making the biochar effect nonsignificant. We concluded that appropriate biochar addition could maintain or enhance the mechanical performance of Portland cement composites, and future research should explore the mechanisms of biochar effects on the performance of cement composites.

**Graphical Abstract:**

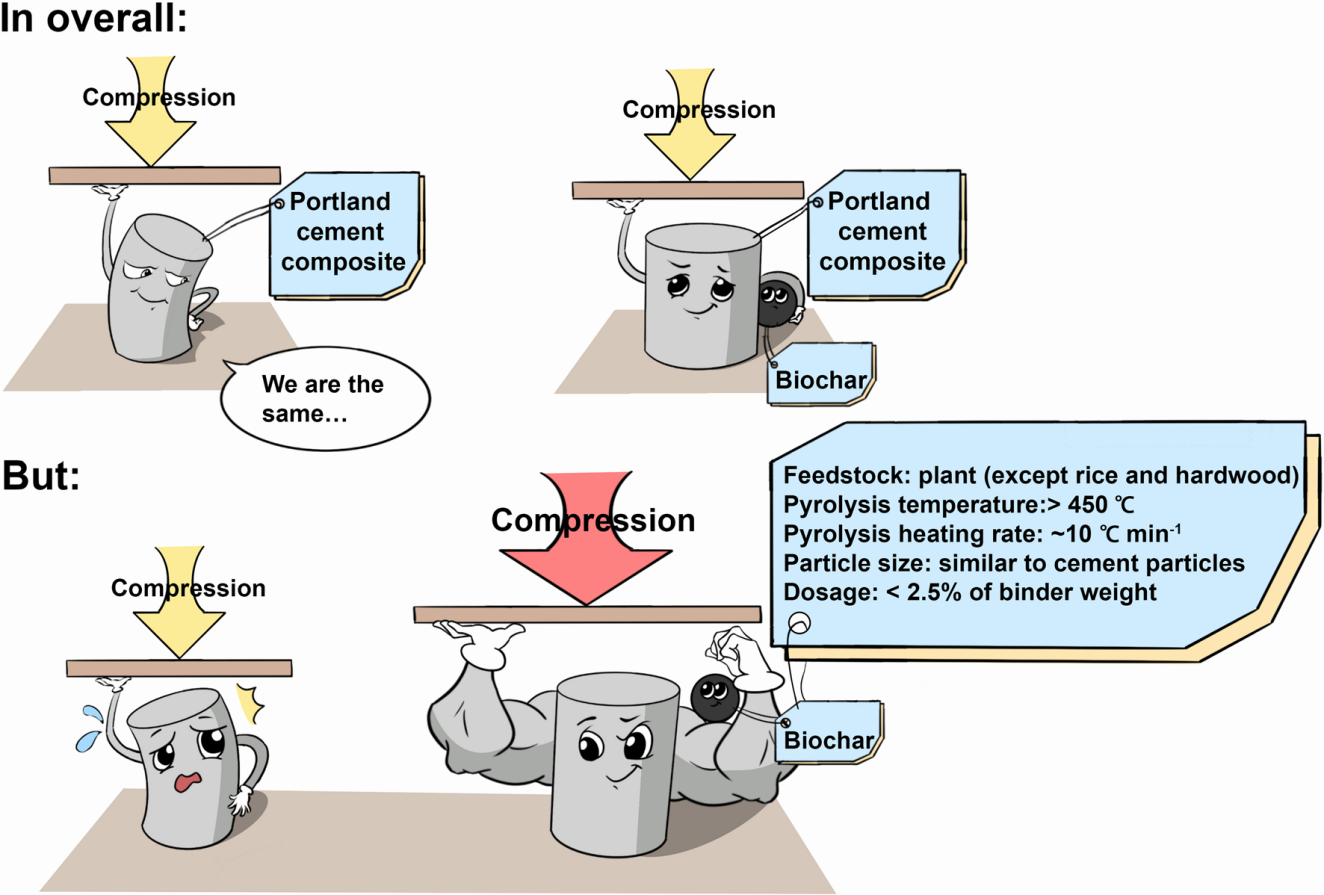

**Supplementary Information:**

The online version contains supplementary material available at 10.1007/s42773-024-00309-2.

## Highlights

- Biochar effects on Portland cement composites were studied through a meta-analysis.
- Effects of biochar production condition, modification and pre-treatment were studied.
- The above parameters affected the compressive strength of Portland cement composites.
- Biochar addition effects were dependent on batch designs of Portland cement composites.

**Supplementary Information:**

The online version contains supplementary material available at 10.1007/s42773-024-00309-2.

## Introduction

Anthropogenic emissions of CO_2_ have resulted in one of the most devastating environmental problems in the twenty-first century (Lamb et al. 2021; Olivier 2022). Efforts are being taken to enhance carbon sinks in terrestrial ecosystems, including urban and other highly human-influenced environments, to attain carbon neutrality and mitigate climate change (Wang et al. 2021). After fossil fuel usage and land-use change, cement production is one of the most significant anthropogenic carbon emissions, accounting for almost 5% (4000 Mt) of global anthropogenic CO_2_ emissions in 2019 (Andrew 2019; Friedlingstein et al. 2022). Replacing cement with supplementary cementitious materials (SCMs), such as fly ash, silica fume and waste glass, could help reduce anthropogenic CO_2_ emissions and improve concrete performance (Li et al. 2022; Mehta and Ashish 2020; Miller et al. 2021). However, traditional SCMs are industrial by-products that will be less available in the future, and these materials do not contribute to carbon removal. Therefore, novel SCMs need to be designed to fill the potential gap.

Adding biochar to Portland cement composites (a general term for cement paste, mortar, and concrete in this article) has emerged as a potential solution to providing the needed SCMs and removing excessive carbon from the atmosphere. Biochar is a stable porous pyrolytic material produced from feedstocks such as waste plant materials and industrial sludge, reducing the release of waste and pollutants (Amalina et al. 2022; Chen et al. 2019). Biochar can improve soil physical and chemical properties, mitigate greenhouse gas emissions, and remediate soil pollution, among other benefits (He et al. 2022; Osman et al. 2022; Singh et al. 2022). Using biochar as SCMs in civil engineering is an emerging field supported by promising results (Danish et al. 2021; Singhal 2023; Tan et al. 2021). For instance, biochar addition to Portland cement composites has shown to promote heat evolution of hydration (Sikora et al. 2022; Zhang et al. 2022a, b, c). Adding wood-derived biochars at 0.5 and 2% (by weight) of cement improved 28-day compressive strength by 16 and 9%, respectively, and decreased the water permeability of concrete by 40% at a dosage of 2% (Gupta et al. 2020b). These results were consistent with the observed dense interfacial transition zone (ITZ) between biochar and cement paste, suggesting that biochar promoted the hydration process (Dixit et al. 2019). The internal curing of biochar assisted the cement hydration process by absorbing and releasing water (Gupta and Mahmood 2022). Biochar addition may also improve concrete thermal conductivity and electromagnetic shielding capacity (di Summa et al. 2023; Ryms et al. 2022). Biochar addition increased the carbon sequestration of Portland cement composites without significantly increasing the composite production cost (Gupta et al. 2018c; Praneeth et al. 2020). Overall, adding biochar to Portland cement composites could improve their performance and increase the carbon sink to mitigate climate change.

Some studies, however, reported adverse effects of biochar addition on the performances of Portland cement composites. For example, Akhtar and Sarmah (2018a, b) found that adding litter-derived biochar decreased 28-day compressive strength, which can be attributed to reduced hydration products due to the dilution effect of biochar addition.  The type and production conditions of biochar also affect the potential of biochar to enhance the performance of Portland cement composites. For instance, biochars produced from barley straw performed better than manure biochars in improving the 28-day compressive strength of concrete (Zhang et al. 2022b), and adding biochars produced at high pyrolysis temperatures resulted in higher compressive strength of cement mortar than biochars produced at low pyrolysis temperatures (Gupta and Kua 2018), demonstrating that biochar properties would significantly affect the performance of Portland cement composites.

Several review papers have discussed biochar effects on Portland cement composites’ mechanical properties (Maljaee et al. 2021a; Senadheera et al. 2023; Zhang et al. 2022a, b, c), but these papers did not review all related literature on this topic, which may cause sampling error and imprecise conclusions. In addition, comprehensive evaluations of biochar addition effects are needed to reconcile contradictory results of biochar effects on the mechanical properties of Portland cement composites. Meta-analysis collects an extensive data set from individual studies on a particular research topic to assess the overall effect numerically and boost the generalization of the collected data; meta-analysis has been widely used in ecology and medicine (Arnqvist and Wooster 1995; Hartung et al. 2008; Hernandez et al. 2020). A recent meta-analysis focused on the effects of cement and aggregate replacement on the mechanical performance of Portland cement composites (Anwar et al. 2022); however, it did not provide details on the properties of the mixtures, especially for biochar, making it difficult to optimize the selection of the mixtures. Therefore, it is necessary to conduct further research to explore and provide quantitative evidence on how biochar properties affect the mechanical performance of Portland cement composites and provide guidance for biochar selection for further research and industrial applications.

We used meta-analysis to quantify the effect of biochar addition on the mechanical performance of Portland cement composites based on 606 paired observations from 51 peer-reviewed papers. The effect size was calculated using 7- and 28-day compressive strengths, which are crucial quality indices for the performance of Portland cement composites and correlated with other performance indicators, such as flexural strength, hardened density, and water permeability (Kosmatka and Wilson 2011). This study considered biochar pyrolysis conditions, including pyrolysis temperature, heating rate and residence time, biochar pre-treatment and modification, biochar dosage, concrete curing type, and cementitious matrices. We hypothesized that: (1) the effect of biochar addition on the performance of Portland cement composites is influenced by biochar pyrolysis condition; (2) biochar pre-treatment and modification impact the effects of biochar addition; (3) the Portland cement composite batch design, including biochar dosage, curing type and forms of composite, influences the effect of biochar addition. This study aims to provide quantitative evidence of the effects of biochar production conditions and its properties and Portland cement composite batching designs on compressive strength with illustrations of the potential mechanisms of biochar addition effects.

## Methods

### Literature search

The data of 7- and 28-day compressive strengths of Portland cement composites for this study were collected from peer-reviewed research papers via Web of Science and Scopus using the following search terms: “biochar” AND “cement” AND “compressive strength.” Papers related to Portland cement composites used as building materials were shortlisted by reviewing the titles and abstracts, with papers on soil and environmental remediation excluded from further data collection and analysis. A total of 387 papers were initially screened; they were filtered to include those that measured 7- and 28-day compressive strengths by scanning abstracts and figures in each paper. This filtration excluded some papers where: (1) biochar was not the only SCMs; (2) microbes were introduced to biochars; (3) biochars were not produced through pyrolysis; (4) incomplete statistical data; (5) Portland cement used in the study was not ordinary Portland cement. Finally, 51 papers were included for the meta-analysis based on papers published before December 1st, 2023, with 41 and 48 papers including 7- and 28-day compressive strength, with 254 and 352 paired observations, respectively (Additional file 1: Table S1). Data from collected papers were organized as paired-observation datasets. Each paired observation was treated as one record. Pyrolysis conditions, biochar properties, and Portland cement composite batching design information were extracted from the literature.

### Data compilation

The means, standard errors/deviations (SE/SD), and the number of replicates (n) for 7- and 28-day compressive strength were extracted from each reference. The compressive strength units were megapascal (MPa), and both control and treatment groups were recorded. All SE were converted into SD via the equation: $SD = SE*\sqrt n$. Data with missing SE/SD were less than 15% of the total data. They were estimated using an imputation method, where the weighted average of SD from the other records was used to estimate the imputed SD (Bracken 1992). Data presented in figures in the literature were extracted through the OriginPro software.

Biochar feedstocks were categorized into plant and organic waste groups. The plant group includes agricultural and forestry plant materials, and the organic waste group includes sludge and manure. Main food crops and wood were selected as they are primary sources of biochar. Some  feedstocks (including wood materials, which were not indicated as hardwood or softwood) were unknown or had a small sample size (for example, bamboo, bagasse, and peanut), and they were categorized as “Other plant materials.” In addition, several feedstock materials from the same origin (including corn, rice, and wheat) were combined as one feedstock source to satisfy the sample size requirement for meta-analysis. Pyrolysis temperature was divided into four groups: “< 350,” “350–450,” “450–550,” and “> 550” °C. The pyrolysis heating rate was divided into three groups: “0–5,” “5–10,” and “> 10” °C min^−1^. For pyrolysis residence time (min), this analysis used three groups of “< 60,” “60–180,” and “> 180” to represent short, medium and long residence  time, respectively. Biochar pre-treatments were categorized into only physical and chemical treatments. Reducing the particle size was the primary physical modification, including using ball milling, sieving and manual grinding, which was treated as grinding. Chemical treatments were recorded as what the papers used.

The factors considered for mixture design were biochar dosage, concrete curing type and cementitious matrices. Biochar dosage was calculated as the ratio of the biochar weight and the binder (cement + biochar) weight, shown as “% of binder weight" or “to binder weight.” Curing type was divided into “carbonization,” “seal,” “dry,” and “wet,” where “ Carbonization” represented curing the composites in the environment with a high concentration of CO_2_ “seal” represented blocking the composites away from the external environment during curing “dry” represented curing the composites under ambient environment; “wet” represented curing the composites under high humidity or submerged environment. Cementitious matrices represented the forms of Portland cement composites, divided into “cement paste,” “mortar,” and “concrete.”

### Data analysis

The meta-analysis used a log-transformed ratio to analyze the effect size of performance parameters by biochar and batching variables (Chen et al. 2022a, b, c; Hedges et al. 1999). Each collected paper was treated as having homogeneous experiment conditions. The individual effect size was calculated according to Eq. (1):

1 $$L = lnRR = ln\left( {\frac{{\overline{X}_{t} }}{{\overline{X}_{c} }}} \right)$$

where $\overline{X}_{t}$ is the mean of the treatment group, which   added biochar into cementitious matrices; $\overline{X}_{c}$ is for the control group without biochar addition. Positive values of *L* or *lnRR* represent an increase of compressive strength compared to the control group and vice versa. Then, the variance of individual effect size was calculated using Eq. (2):

2 $$v = \frac{{S_{t}^{2} }}{{n_{t} *\overline{X}_{t}^{2} }} + \frac{{S_{c}^{2} }}{{n_{c} *\overline{X}_{c}^{2} }}$$

where *S*_*t*_ and *S*_*c*_ are standard deviations of the treatment and control groups; *n*_*t*_ and *n*_*c*_ are sample sizes of the treatment and control groups, respectively. Considering the effect of sample size and variance, a weighted mean of effect size for each categorized parameter was calculated to obtain an overall effect response of each factor (Eq. (3)):

3 $$L_{w} = \frac{{\sum\nolimits_{i = 1}^{k} {w_{i} } *L_{i} }}{{\sum\nolimits_{i = 1}^{k} {w_{i} } }}$$

where *k* is the number of paired data points; *w*_*i*_ is the weighting factor, which is sensitive to *v*_*i*_ (Hedges et al. 1999). After the weight effect size was calculated, a 95% confidential interval (95CI) was calculated using Eq. (4):

4 $$95CI = L_{w} \pm 1.96*se_{{L_{w} }}$$

where $se_{{L_{w} }}$ is the standard error of *L*_*w*_. When 95CI does not overlap 0, the effect is significant. To intuitively illustrate the effect, we back-transformed the log response ratio to a natural response ratio in percentage using Eq. (5) and called it as an effect index:

5 $$Effect\,index\,(\% ) = \left( {e^{lnRR} - 1} \right)*100$$

The meta-analysis was processed using the metagear package in R, and all correlation analyses in this study were simple linear regressions conducted using ggplot2 in R, with the following equation (Eq. (6)):

6 $$L = lnRR = \beta_{0} + \beta_{1} *x + \varepsilon$$

where *β*_1_ is for coefficient; *x* is the factor; ε is the sampling error. Based on the database size in this study, the regression will only be used for factors with at least 100 paired comparisons.

## Results and discussion

Overall, biochar addition to Portland cement composites did not reduce the 7- and 28-day compressive strengths (Additional file 1: Fig. S1). Moreover, the effect sizes of 7- and 28-day compressive strengths were positively correlated (R^2^ = 0.72,* p *< 0.01) (Additional file 1: Fig. S2), indicating that biochar addition would maintain its effect on compressive strength throughout the curing process. However, as the overall effect sizes for 7- and 28-day compressive strengths had high heterogeneities (*p* < 0.01 for both parameters), the potential of biochar to maintain the compressive strength of Portland cement composites varied significantly with biochar type and pyrolysis condition, as well as the batching design.

### Effects of biochar characteristics

#### Feedstocks

**Fig. 1:**
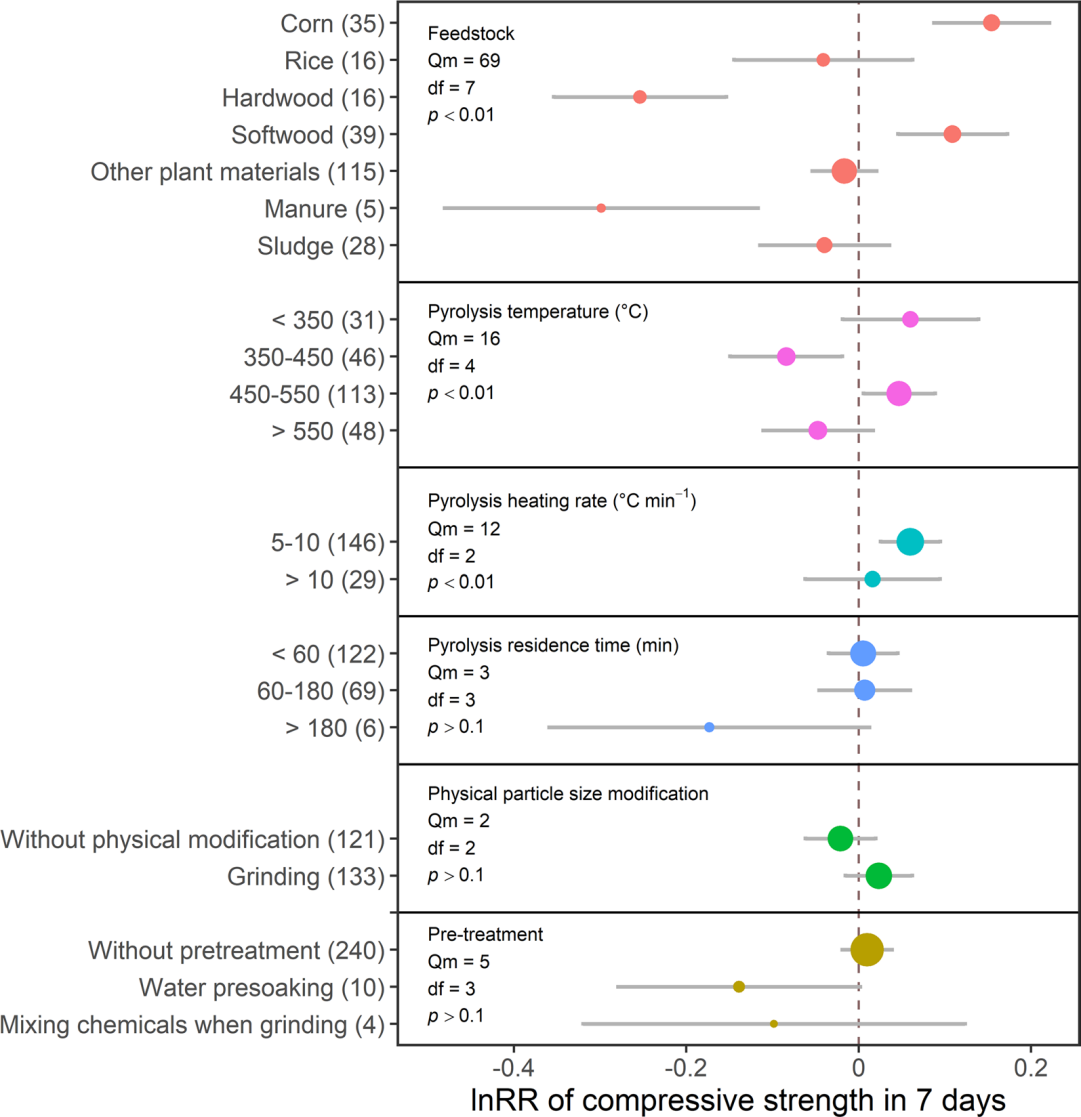
The effect sizes of biochar addition on the 7-day compressive strength of Portland cement composites, as affected by the feedstock used for biochar production, pyrolysis temperature, pyrolysis residence time, pyrolysis heating rate, biochar modification and pre-treatment. Each point represents effect sizes, and the size of the point represents the relative number of records compared to the total records. Grey bars represent 95CI. The vertical dash line represents the value of 0. The numbers of records are indicated in the brackets

**Fig. 2:**
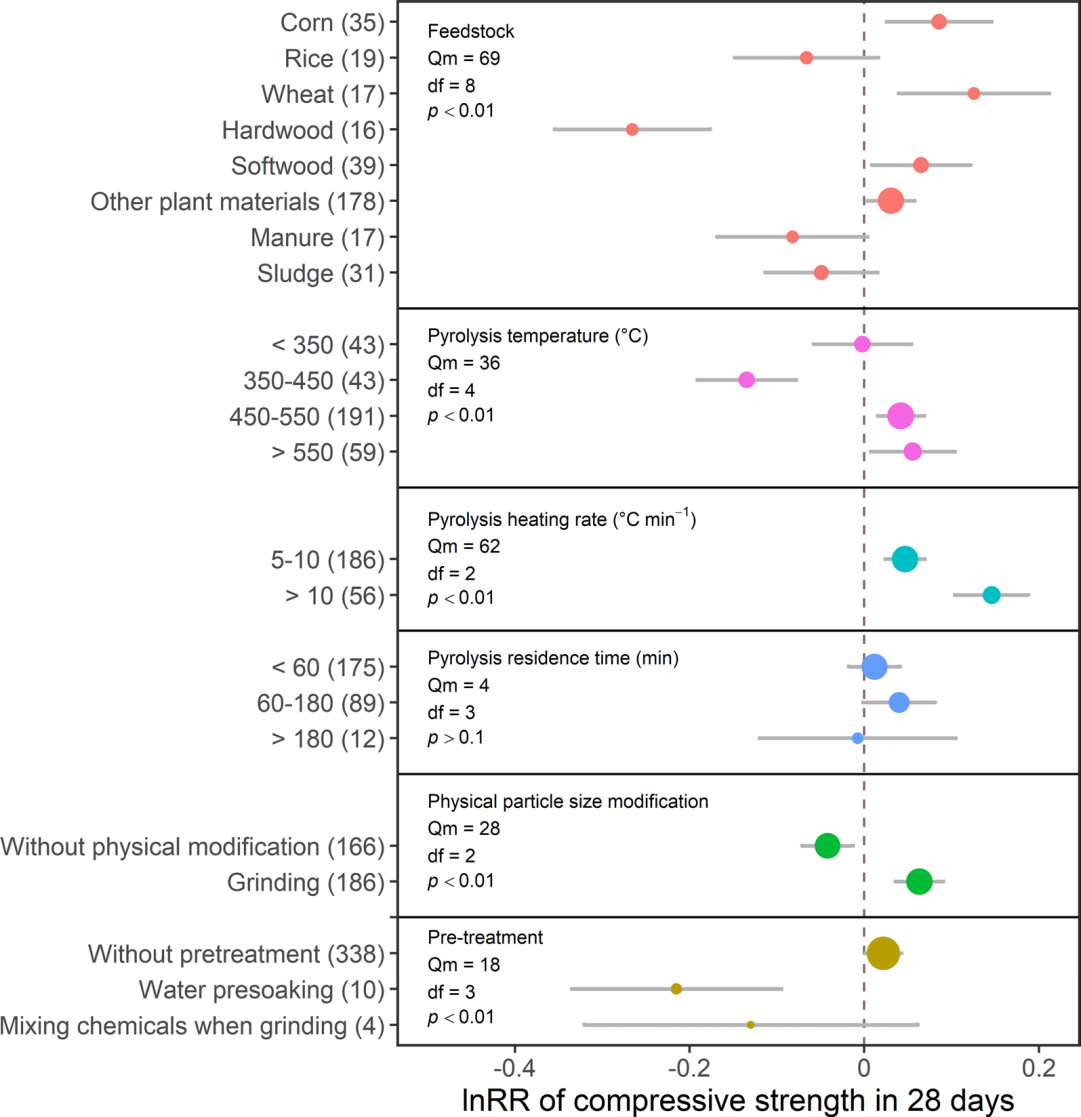
The effect sizes of biochar addition on the 28-day compressive strength of Portland cement composites, as affected by the feedstock used for biochar production, pyrolysis temperature, pyrolysis residence time, pyrolysis heating rate, and biochar modification and pre-treatment. Each point represents effect sizes, and the size of the point represents the relative number of records compared to the total records. Grey bars represent 95CI. The vertical dash line represents the value of 0. The numbers of records are indicated in the brackets

The effect of biochar produced from different feedstocks on the compressive strength of Portland cement composites was inconsistent. In particular, biochar produced from corn significantly increased the 7-day compressive strength by 17% (Fig. 1, Table 1), whereas most plant-based biochar types increased the 28-day compressive strength by 3–13% (Fig. 2, Table 2). However, biochars produced from rice residues did not affect the compressive strength after 7 or 28 days. Meanwhile, biochars produced from forestry materials exhibited contradictory effects, as softwood biochars increased the 7- and 28-day compressive strength by 12 and 7%, respectively, while hardwood biochars decreased them by 22 and 23%, respectively (Figs. 1 and 2; Tables 1 and 2). Biochars produced from manure decreased the 7-day compressive strength by 26%; however, this effect was minimal after 28 days. Finally, biochars produced from sludge did not increase or decrease either of the compressive strengths of Portland cement composites.

**Table 1:** Effect indexes and critical properties of biochar used for 7-day compressive strength measurements, including means and numbers of records

Biochar variables	Effect index (%)		C content (%)		Molar O/C ratio		Specific surface area (m^2^ g^−1^)		Si concentration (%)		SiO_2_ concentration (%)	
	Mean	n	Mean	n	Mean	n	Mean	n	Mean	n	Mean	n
Feedstock												
Corn	17	35	–	–	–	–	483	24	–	–	4.6	24
Rice	− 4	16	43	11	1.02	7	17	9	5.67	7	66.4	9
Hardwood	− 22	16	66.7	9	0.24	1	60	8	–	–	–	–
Softwood	12	39	76	29	0.17	29	147	9	0.42	29	15.4	3
O.P.	− 2	115	68	72	0.29	50	62	49	0.36	28	23.2	23
Manure	− 26	5	19	5	3.06	5	–	–	0.03	5	–	–
Sludge	− 4	28	47	12	0.89	12	250	5	0.34	6	–	–
Pyrolysis temperature (ºC)												
< 350	6	31	60	17	0.33	17	403	9	0.40	16	4.5	6
350–450	− 8	46	56	24	1.19	16	221	15	0.15	6	19.8	12
450–550	5	113	69	71	0.35	59	164	61	1.17	46	22.7	30
> 550	− 5	48	67	17	0.69	11	27	18	0.21	6	34.6	9
Pyrolysis heating rate (ºC min^-1^)												
5–10	6	146	70	85	0.28	79	217	75	0.37	58	13.2	32
> 10	2	29	51	6	0.94	6	118	4	9.70	4	44.2	6
Pyrolysis residence time (min)												
< 60	1	122	64	94	0.55	74	84	47	0.95	62	34.1	26
60–180	1	69	65	22	0.37	22	288	45	0.40	5	4.6	24
> 180	− 16	6	–	–	–	–	142	2	–	–	–	–

The Si concentration represents the Si content to the total weight of biochar, with the Si content typically determined through inductively coupled plasma spectroscopy. The SiO_2_ concentration represents the SiO_2_ content to the total weight of oxides, with the SiO_2_ content typically determined through X-ray fluorescence. The term “Effect index” is defined by Eq. (5)

*O.P.* other plant materials. *n* numbers of records

**Table 2:** Effect indexes and critical properties of biochar used for 28-day compressive strength measurements, including means and numbers of records

Biochar variables	Effect index (%)		C content (%)		Molar O/C ratio		Specific surface area (m^2^ g^−1^)		Si concentration (%)		SiO_2_ concentration (%)	
	Mean	n	Mean	n	Mean	n	Mean	n	Mean	n	Mean	n
Feedstock												
Corn	9	35	–	–	–	–	483	24	–	–	4.6	24
Rice	–6	19	47	14	0.81	10	19	7	0.25	5	49.3	14
Wheat	13	17	67	11	0.34	5	103	12	–	–	10.7	5
Hardwood	− 23	16	66.7	9	0.24	1	60	8	–	–	–	–
Softwood	7	39	76	29	0.17	29	147	9	0.42	29	15.4	3
O.P.	3	178	73	112	0.25	68	82	73	0.40	39	18.5	30
Manure	− 8	17	19	5	3.06	5	–	–	0.03	5	–	–
Sludge	− 5	31	47	12	0.89	12	250	5	0.34	6	–	–
Pyrolysis temperature (ºC)												
< 350	0	43	60	17	0.33	17	403	9	0.40	16	4.5	6
350–450	− 13	43	51.7	18	1.73	10	279	11	0.15	6	4.7	6
450–550	4	191	70	124	0.30	91	150	88	0.38	55	19.9	53
> 550	6	59	70	28	0.69	11	61	29	0.21	6	34.6	9
Pyrolysis heating rate (ºC/min)												
5–10	5	186	70	124	0.29	102	221	89	0.37	58	14.6	55
> 10	16	56	71	18	1.62	2	54	28	–	–	44.2	6
Pyrolysis residence time (min)												
< 60	1	175	66	132	0.48	96	102	69	0.36	61	25.8	49
60–180	4	89	69	42	0.31	32	240	55	0.40	5	4.6	24
> 180	− 1	12	69	6	–	–	172	8	–	–	–	–

The Si concentration represents the Si content to the total weight of biochar, with the Si content typically determined through inductively coupled plasma spectroscopy. The SiO_2_ concentration represents the SiO_2_ content to the total weight of oxides, with the SiO_2_ content typically determined through X-ray fluorescence. The term “Effect index” is defined by Eq. (5)

*O.P.* other plant materials. *n* numbers of records

The superiority of biochars produced from agricultural feedstock over other feedstock types in increasing the compressive strength of Portland cement composites could be attributed to their low molar oxygen/carbon (O/C) ratio, primarily caused by the low carbon contents of biochars (Tables 1 and 2), as lower molar O/C ratio is associated with higher biochar hydrophobicity due to its low content of oxygen-containing functional groups (Hassan et al. 2020; Xing et al. 2019; Zhao et al. 2013). Other researchers found that hydrophobic silica fume could accelerate cement hydration due to more available water surrounding cement particles, offsetting the negative effect of the larger particle size of the hydrophobic silica fume, indicating accelerated cement hydration under high hydrophobicity (Jeong et al. 2020). Biochars produced from plant wastes had lower molar O/C ratios than manure and sludge biochars (Tables 1 and 2), contributing to their high hydrophobicity and potential for enhancing cement hydration. However, agriculture-sourced biochars increased 7- and 28-day compressive strengths, but forestry-sourced biochars did not, even though forestry-sourced biochars had higher carbon contents (76 and 76%, respectively in 7- and 28-day compressive strength) than agriculture-sourced biochars (43 and 56%, respectively) (Tables 1 and 2). As forestry-sourced biochars had a more macroporous structure than agriculture-sourced biochars due to their high lignin content (El-Naggar et al. 2022), agriculture-sourced biochars have a highly mesoporous structure, which may contribute to considerable water-absorption-release capacity, where biochars absorb water in the early curing stage to densify the cementitious matrix and then desorb water in response to a humidity gradient to maintain cement hydration (Khan et al. 2022), increasing the compressive strength. However, due to the lack of data, it is not possible to conclude the different effects between hardwood and softwood biochar addition. More research is needed to better understand the mechanisms involved.

Ash content, which includes oxides, could also affect cement hydration. Amorphous silica oxide (SiO_2_) was the most critical oxide for cement hydration, contributing to the pozzolanic reaction, in which SiO_2_ would consume Ca(OH)_2_ to form calcium silicate hydrate (C–S–H) to enhance the growth of strength (Thomas 2011; Zhang et al. 2020; Zhou et al. 2020). Other oxides, such as Fe_2_O_3_ and Al_2_O_3_, can negatively and positively, respectively, affect cement hydration (Stephan et al. 2008). In this study, biochars produced from plant sources had a relatively high Si concentration (0.4% on average). The SiO_2_ can make up most of the oxides (19% on average) in the 28-day compressive strength measurement. In contrast, manure biochars contained the least Si, causing the least positive effect from the pozzolanic reaction (Table 2). These results illustrate that the positive effect of Si on 28-day compressive strength was better than other types of biochar. Similarly, Si and SiO_2_ contents of biochars produced from plant sources for 7-day compressive strength measurement were the highest compared to other biochars (1 and 22% on average, respectively; Table 1), indicating relatively intense pozzolanic reactions during early cement hydration.

Overall feedstock effects on compressive strength demonstrated that biochars produced from plant wastes (except rice) had significant positive effects, and manure biochars had significant adverse effects on 7-day compressive strength, as manure biochars had a higher molar O/C ratio than the plant-based biochars. Meanwhile, elements and oxide content, especially for Si, which contributed to the pozzolanic reaction, could promote cement hydration. However, more data  are required to analyze the effect of feedstock type and oxides on cement hydration to validate the above findings.

#### Pyrolysis condition

Adding biochars produced at pyrolysis temperatures between 450 and 550 °C significantly improved 7-day compressive strengths by 5%, but biochars produced between 350 and 450 °C decreased this parameter by 8% (Fig. 1, Table 1).  In addition, on one hand, biochars produced at pyrolysis heating rates between 5 and 10 °C min^−1^ improved 7-day compressive strength by 6%. On the other hand, adding biochars produced at a temperature higher than 450 °C significantly improved 28-day compressive strength by more than 4%, and biochars produced at higher heating rates more significantly increased 28-day compressive strength compared to lower rates (Fig. 2, Table 2). However, pyrolysis residence time did not affect the compressive strengths of Portland cement composites.

**Fig. 3:**
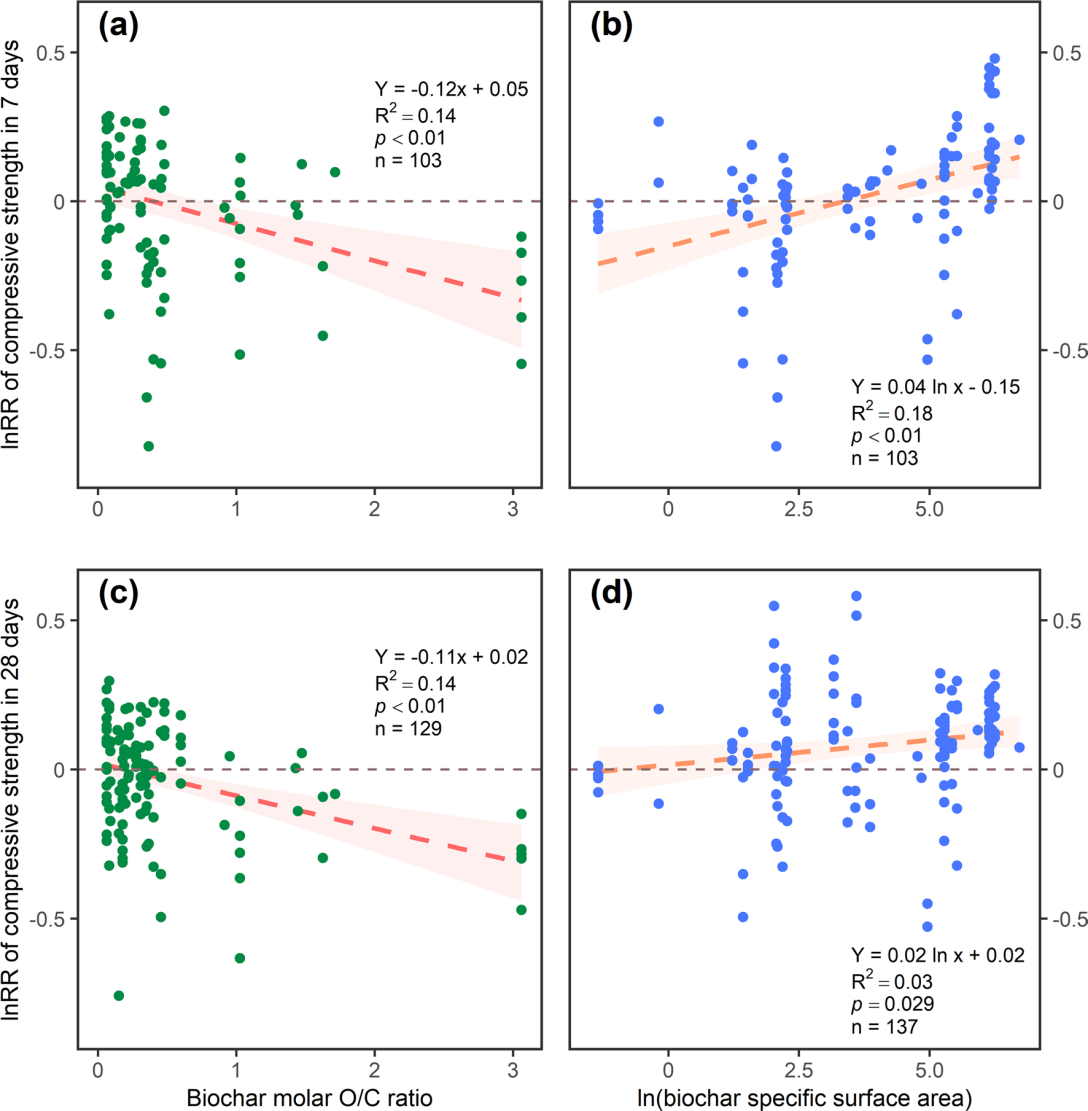
Linear correlations between (a) the molar O/C (oxygen/carbon) ratio of biochar and effect size of 7-day compressive strength, (b) the natural log-transformed specific surface area of biochar and effect size of 7-day compressive strength, (c) the molar O/C ratio of biochar and effect size of 28-day compressive strength, and (d) the natural log-transformed specific surface area of biochar and effect size of 28-day compressive strength. Points in each figure represent paired records. The simple linear regression lines with 95% confidential intervals are shown, with the number of records (n) presented. The horizontal dash lines represent the value of 0

Pyrolysis temperature and heating rate highly affected biochar properties, including molar O/C ratios and specific surface areas. In this study, both compressive strengths negatively correlated to the molar O/C ratio of biochars, while positively correlated to the specific surface area of biochars (Fig. 3). Biochars produced at temperatures between 450 and 550 °C had a relatively lower molar O/C ratio (Tables 1 and 2), as higher temperature conditions would decompose organic substances, increasing C content and decreasing O content, resulting in a decreased molar O/C ratio (Ghodake et al. 2021). However, the molar O/C ratio of biochars produced at 350 °C was similar to biochars produced between 450 and 550 °C with different effects. In this study, most of the biochars produced below 350 °C were forestry-sourced biochars with high lignin contents, leading to a relatively low molar O/C ratio, while other temperature categories comprising other feedstocks with relatively low C content (Table 1 and 2). Meanwhile, biochars with high specific surface areas, such as biochars produced at temperatures < 350 °C and between 450 and 550 °C in this study (Tables 1 and 2), could provide more nucleation sites for cement hydration, contributing to more hydration products (including C–S–H) to increase compressive strength (Restuccia and Ferro 2016; Zhang et al. 2022a, b, c). However, organic matter would be left in biochars produced at low temperatures due to uncompleted decomposition, such as fatty acids and residual saccharides (Chen et al. 2022a, b, c; Das et al. 2021; Gupta et al. 2020a, 2020c; Muthukrishnan et al. 2019). These organic matters would retard cement hydration (Choi and Choi 2021; Kochova et al. 2017), counteracting the benefits of the high specific surface area. In this case, biochars produced at a lower temperature did not improve compressive strength. However, excessive pyrolysis temperature (> 500 °C) could damage the biochar pore structure to break water absorption and release capacity, which was a counterproductive effect (Fu et al. 2012), and this finding could indicate the insignificant effects of high-temperature (> 550 °C) biochar addition on 7-day compressive strength. As for the pyrolysis heating rate, rates between 5 and 10 °C min^−1^ had a relatively high specific surface area and low molar O/C ratio in 7- and 28-day compressive strength measurements (Tables 1 and 2). Slow pyrolysis (heating rate < 50 °C min^−1^) would result in a higher biochar yield. In contrast, fast pyrolysis would produce more oil and gas phases due to secondary reactions of decomposed polysaccharides, reducing the yield of the solid phase (Al-Rumaihi et al. 2022; Chen et al. 2021; Ghodake et al. 2021). This information indicated that the mild pyrolysis heating conditions would retard biomass gasification and liquefication, where biochars could maintain their structure of carbon skeleton to benefit cement hydration.

The overall effect of pyrolysis condition on 7- and 28-day compressive strengths illustrated that high pyrolysis temperature would improve 7- and 28-day compressive strengths. Additionally, medium heating rates could significantly improve the 28-day compressive strength, which was highly negatively correlated to biochar molar O/C ratio and positively correlated with specific surface area. It is necessary to enlarge the research range to include biochars produced under different conditions to select optimal biochars for altering the performance of Portland cement composites.

#### Biochar modification and pre-treatment

Grinding was the primary physical modification of reducing particle size; most grinding was done through ball milling. Biochar grinding did not reduce the 7-day compressive strength but increased the 28-day compressive strength by 7% (Figs. 1 and 2). However, biochars without physical modification will decrease the 28-day compressive strength. Biochar grinding could reduce the biochar particle sizes (D_90_ is around 45 µm), similar to or smaller than the cement particles (D_90_ is around 40 µm), compared to biochars without this modification (D_90_ is around 200 µm) (Additional file 1: Tables S2 and S3). Such tiny particles could fill the ITZ between cement particles and aggregates as a filler and improve the Portland cement composites’ compressive strengths as nucleation sites due to the enlarged specific surface area (Dixit et al. 2019; Gupta et al. 2020a, b, c; Yang and Wang 2021). In the early curing stage, the reduced water/binder ratio, by water absorption of biochars, had a higher effect than the filler effect, and water would be desorbed later due to the humidity gradient (Gupta 2021), maintaining the cement hydration. In the later curing process, most cement particles reacted, and the filler effect was superior to the water absorption and release effect. Research on carbon nanotubes, which were nano-size carbon materials that could considerably increase concrete compressive strength, could also provide valid evidence that tiny particle size would be beneficial to increase compressive strength (Silvestro and Gleize 2020; Zhang et al. 2023). However, as grinding could destroy the original pore structure of biochars, this modification would retard the function of nucleation at the early curing stage. As grinding could destroy macropores with less water absorption and release capacity, biochar’s water-holding capacity and filler effect would increase compressive strength later, offsetting the retardance in the early curing stage.

Presoaking biochars with water and other pre-treatment methods did not affect either compressive strength (Figs. 1 and 2). However, only two studies in the database of this paper reported the effect of pre-treating biochar with water on its potential to enhance compressive strength, which made it hard to evaluate the effect of presoaking biochar. For instance, Gupta and Kua (2018) presoaked biochar with water to provide additional water to mortar, and they found an improvement in 28-day compressive strength. However, Jafari et al. (2023) reported that water-presoaking treatment could not counteract the negative effect of high biochar dosage, but such a decrease could be mitigated by combining the high biochar dosage with other SCMs, such as MgO expansive additives (Mo et al. 2019). Another paper, not included in this meta-analysis, indicated that presoaking biochar could maintain concrete strength in the long term, showing the potential benefit of presoaking biochar (Sirico et al. 2022). Haque et al. (2021) also mixed biochars with stearic acid when grinding to obtain super-hydrophobic surface characteristics. However, the high biochar dosage did not affect the 28-day compressive strength, as further discussed in Sect. 3.2.1. Other biochar pre-treatments were also applied, such as carbon dioxide pre-dosage (Gupta et al. 2018b), melamine pre-treatment (Jeong et al. 2022), alkaline electromagnetic pre-treatment (Beskopylny et al. 2022) and acid pre-treatment (Zeidabadi et al. 2018), which introduce additional substances or oxygen-contained functional groups to biochar surfaces to alter its physical and chemical properties, but they were not included in this article due to the small sample size. The lack of data limited further analysis of pre-treatment effects on the compressive strength of Portland cement composites.

Overall, the effects of biochar modification and pre-treatment methods on compressive strength demonstrated that modifying biochar with an appropriate method could enhance their potential to improve the compressive strength of Portland cement composites. Grinding could improve the 28-day compressive strength due to the filler effect. However, as data on biochar modification and pre-treatments are scarce, future studies need to explore this field to better understand the effects of biochar addition on the performance of Portland cement composites.

### Effects of batching design and curing

#### Batching dosage of biochars

**Fig. 4:**
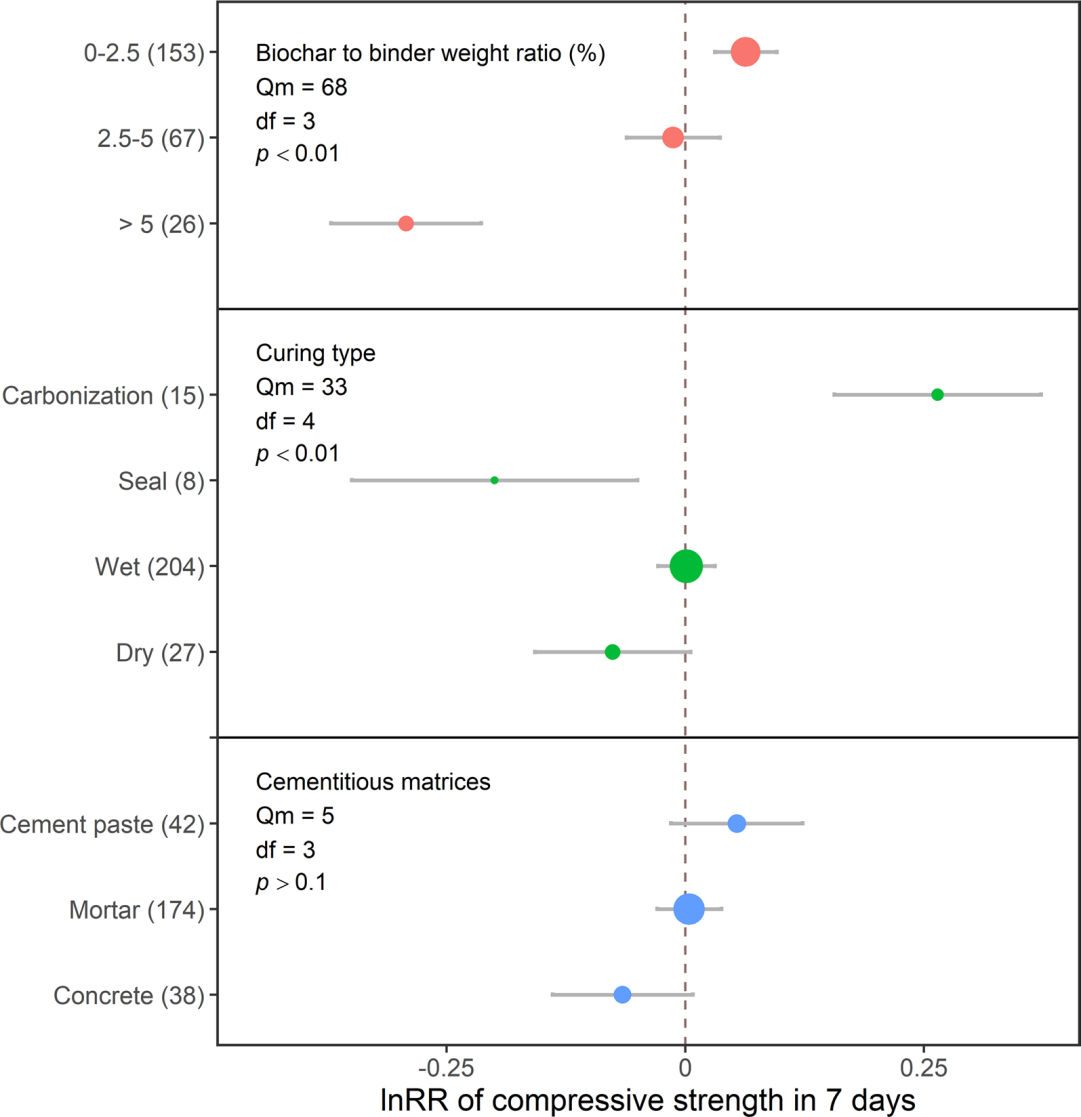
The effect sizes of biochar addition on the 7-day compressive strength of Portland cement composites, as affected by the dosage of biochar application, curing method, and cementitious matrix. Each point represents effect sizes, and the size of the point represents the relative number of records compared to the total records. Grey bars represent 95CI. The vertical dash line represents the value of 0. The numbers of records are indicated in the brackets

**Fig. 5:**
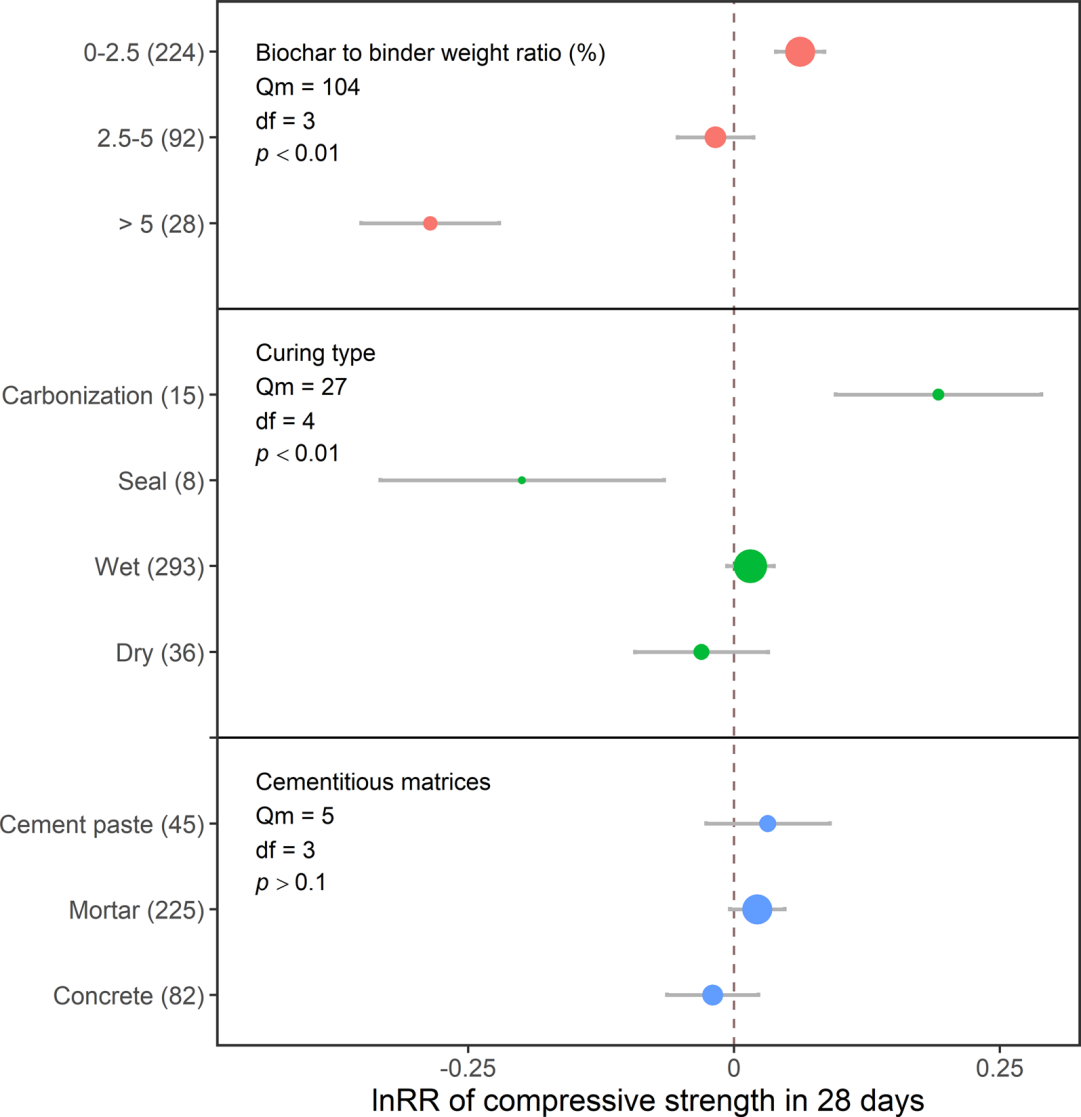
The effect sizes of biochar addition on 28-day compressive strength of Portland cement composites, as affected by the dosage of biochar application, curing method, and cementitious matrix. Each point represents effect sizes, and the size of the point represents the relative number of records compared to the total records. Grey bars represent 95CI. The vertical dash line represents the value of 0. The numbers of records are indicated in the brackets

**Fig. 6:**
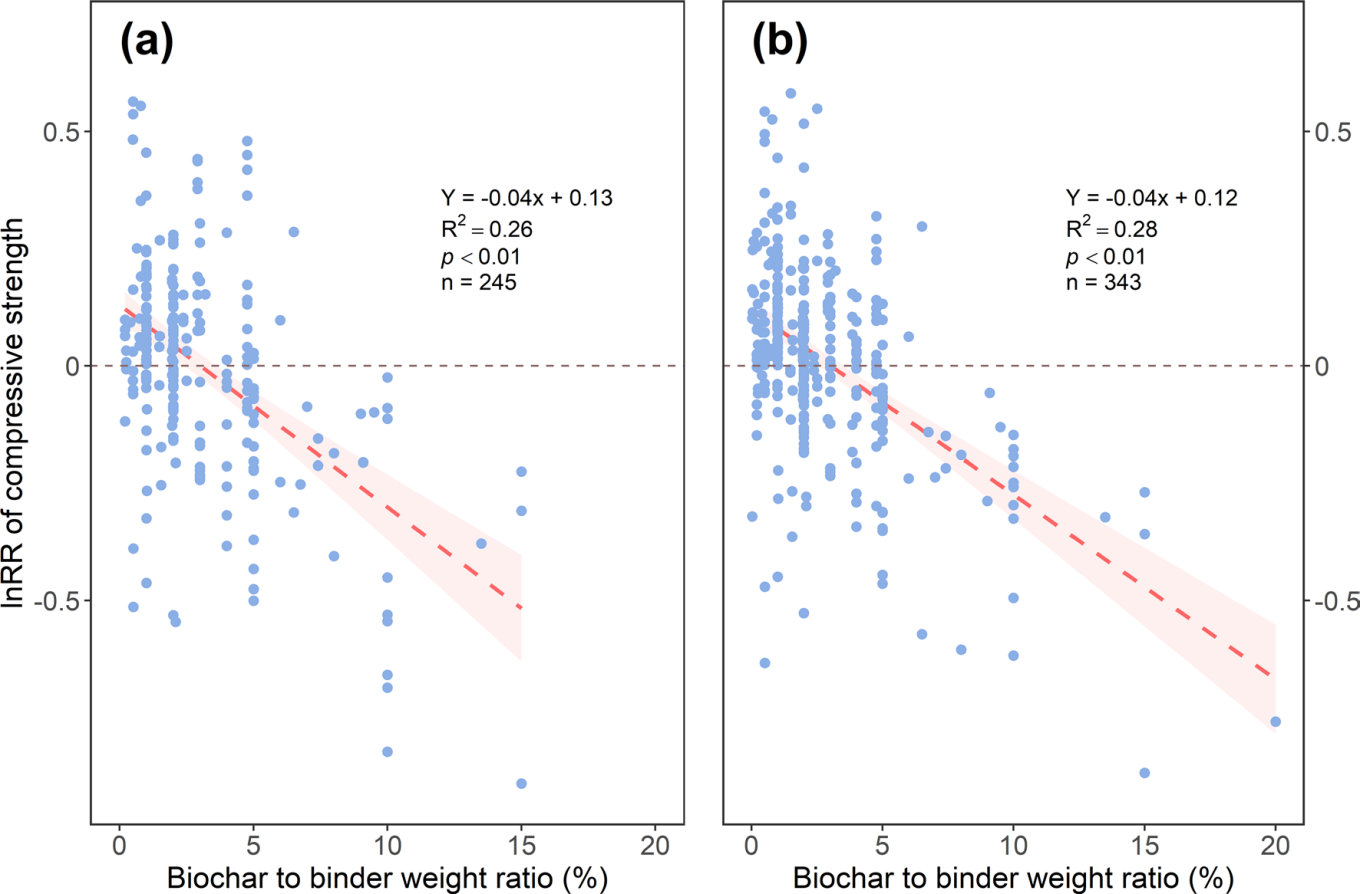
Linear correlations between biochar dosage and the compressive strength of Portland cement composites: (a) biochar to binder weight ratio and effect size of 7-day compressive strength, (b) biochar to binder weight ratio and effect size of 28-day compressive strength. Points in each figure represent paired records. The simple linear regression lines with 95% confidential intervals are shown, with the number of records (n) presented. The horizontal dash lines represent the value of 0

Low biochar dosages (< 2.5% of binder weight) increased 7- and 28-day compressive strengths by 6 and 7%, respectively (Figs. 4 and 5). However, higher biochar dosages negatively impacted compressive strength (Fig. 6) due to its porous structure, which could not strengthen Portland cement composites (Mohan et al. 2014). In addition, higher biochar doses may dilute cement hydration products and cause agglomeration (Mota-Panizio et al. 2023). In particular, biochar would agglomerate through van der Waal's forces when its dosage was more than 5% of cement weight (Gupta et al. 2018a; Maljaee et al. 2021b), indicating that biochar may be poorly dispersed in the cementitious matrix when applied at a higher dosage, leading to a heterogeneous composition and structure of cementitious matrices. Furthermore, excessive biochar dosage would compete with cement to absorb water, which would retard the cement hydration process and strength growth (Tan et al. 2022). However, the adverse effects of biochar at different dosages on compressive strength may also be due to the damaging effects of the pyrolysis process on the biochar pore structure (Zhang et al. 2022b). As mentioned in previous sections, the undecomposed matter left in biochar after pyrolysis would also negatively affect the cement hydration process. Forestry-sourced biochar, containing less Si, might not trade off the dilution effect (Akhtar and Sarmah 2018a, b; Ghodake et al. 2021). Therefore, although a low biochar dosage would increase the compressive strength of Portland cement composites, other factors, including those mentioned above, might offset the low-dosage benefits.

Overall, our results suggest that the optimal biochar dosage to improve the compressive strengths of Portland cement composites is less than 2.5% of binder weight due to the filler effect, nucleation effect and potential pozzolanic reaction. However, other factors might counteract the benefits of low biochar dosage, indicating that careful consideration of biochar addition to Portland cement composites is needed.

#### Cement curing and cementitious matrices

Wet and dry curing, as the most common curing method, did not affect compressive strength. Carbonization curing was the most effective method for improving compressive strength, leading to 30 and 21% increases in 7- and 28-day compressive strengths, respectively (Figs. 4 and 5). Carbonation curing might convert cement composites and hydration products into densified carbonatized composites and silica chains; however, it would consume water, which requires subsequent wet curing to continue the cement hydration process (Chen et al. 2022a, b, c; Liu and Meng 2021). However, this curing method needs to be carefully considered as it requires the use of CO_2_, especially in steel-reinforced concrete; it would destroy the passive layer around steel reinforcement to worsen corrosion and decrease mechanical performance (Kua and Tan 2023; Marques et al. 2013; Tapan and Aboutaha 2011). Sealed curing, on the other hand, had adverse effects, with a 22% decrease in both 7- and 28-day compressive strengths. Sealed curing would physically cover the surfaces of the Portland cement composites to prevent water loss, and the internal curing of biochar would improve compressive strength (Maljaee et al. 2021a, b; Wang et al. 2019). However, the decreased effect of biochar addition in this study indicated that other factors would cooperate with curing, including biochar particle size (Yang and Wang 2021) and dosage (Haque et al. 2021), where the negative effects of particle size and dosage counteracted the positive effect of sealed curing in this study.

Cementitious matrices did not affect compressive strengths (Figs. 4 and 5). It was expected that biochar addition would increase the compressive strength of cement paste, based on the degree of cement hydration, as discussed in previous sections. However, the presence of salts, such as sodium salt and sylvite, in biochar would cover biochar or interact with C–S–H, retarding cement hydration or destroying the C–S–H structure (Gupta et al. 2021; Maljaee et al. 2021a, b; Restuccia and Ferro 2016). These mechanisms counteracted the benefits of biochar addition to cement paste. When coexisting with aggregates, biochar, especially ground biochar with tiny particle sizes close to cement particles, could accelerate hydration and work as fillers to fill the pores of the ITZ between aggregates and cement paste (Park et al. 2021; Scrivener et al. 2004). However, one hypothesis suggested that filling pores might make other tiny particles gather around fine aggregates, providing a convenient route for cracking and decreasing the compressive strength (Aziz et al. 2023). Although requiring validation, such a viewpoint indicated more complex mechanisms of biochar effects, requiring complex models to describe it. In addition, various factors affected mortar and concrete’s compressive strength, including water/binder ratio, coarse aggregate properties, coarse aggregate amount and ITZ properties; effects of these factors indicated that the alternation of cement quality would not significantly affect concrete and mortar’s strength (Maso 1996; Scrivener et al. 2004; Sims et al. 2019). Therefore, studies on the effects of biochar addition to mortar and concrete should address specific composite types, such as lightweight concrete and ultra-high-performance concrete.

Overall curing effects on compressive strength illustrated that carbonation curing would promote the effects of biochar addition. On the other hand, the composition of cementitious matrices did not impact the compressive strength of Portland cement composites, indicating that specializing in matrices in future research is necessary to provide more detailed and precise information on biochar effects on Portland cement composites.

### Limitations and future research perspectives

The lack of some essential details in the literature included in the meta-analysis is one of the major limitations of this study. This limitation may cause uncertainties in the meta-analysis. For instance, misestimation of the variances of parameters is possible due to  the missing SE/SD. In addition, missing information on pyrolysis conditions, including pyrolysis temperature, residence time and heating rate, element content, specific surface area, pore structure, ash and volatile matter contents, and chemical properties of biochars, limited the explanations on the mechanism of the effects and did not enable us to conduct additional correlation analyses, including establishing a structural equation model. Furthermore, due to the lack of data, this study did not include other performances of Portland cement composites, such as flexural strength and durability. In addition, this study did not include papers focusing on a combination of biochar and other SCMs, although some studies illustrated improvements in the mechanical performance of Portland cement composites (Akhtar and Sarmah 2018b; Chen et al. 2022a, b, c; Gupta and Kua 2020), which may limit the application of results in this study. This study also did not include the pre-treatment and modification of feedstocks. Finally, this study only focused on one aspect of the mechanical performance of Portland cement composites, and more research and review should be conducted in the future.

Several aspects need to be addressed in future research: (1) details on biochar production conditions and biochar properties, including pyrolysis conditions, proximate analysis, element contents, and physicochemical properties need to be provided  for further relationship and mechanism analysis and modelling (Song et al. 2023; Zhu et al. 2023); (2) the potential application of engineered or modified biochars need to be explored to potentially increase biochar dosage while maintaining Portland cement composites’ properties; (3) the analysis of batching factors, including cement types, concrete batching type and combining biochar with other SCMs and reinforcement, needs to be conducted to figure out how biochar works in specific Portland cement composites; (4) the measurements for parameters of Portland cement composites need to be extended to quantify the overall effect of biochar addition; (5) the leaching risk of biochar constituents, such as polycyclic aromatic hydrocarbons and heavy metal, needs to be estimated to evaluate the health risks of biochar addition to Portland cement composites (Duan et al. 2019).

## Conclusions

Overall, we  concluded that adding pyrolytic biochars did not decrease the compressive strength of Portland cement composites. Plant-based biochars, rather than organic-waste biochars, are ideal for addition to Portland cement composites. We recommend that biochars should be produced at high temperatures (> 450 °C) with a slow pyrolysis rate (around 10 °C min^−1^) to optimize the positive effects of biochars on Portland cement composites. The reduced particle size of biochars, at least similar to cement particle size (D_90_ is around 40 µm), is recommended to accelerate the cement hydration process; water-presoaking of biochars provide more available water for cement hydration, but more research is required to valid the benefits. The low molar O/C ratio and high specific surface area of biochars were highly correlated to the improvement effects of biochar addition, which were substantially affected by biochar feedstock type, pyrolysis condition and pre-treatment. On the other hand, low biochar dosages (< 2.5% of binder weight) improved compressive strength. Biochars also cooperated with aggregates to affect compressive strength, but the performance of biochar addition on concrete and mortar is highly context-specific due to the complexity of compositions and properties of aggregates. More mechanistic research and modelling, review of environmental issues, including carbon sequestration, life-cycle issues, and leakage of toxic substances, are necessary to further understand the impact of biochar addition on the concrete industry.

### Supplementary Information

**Additional file 1: Table S1.** List of papers used for meta-analysis with the parameters of 7- and 28-day compressive strength. **Table S2.** Particle size distribution (mean ± standard deviation) of ground and original biochars used for testing 7-day compressive strength. D_10_, D_50_ and D_90_: the maximum diameter containing 10%, 50% and 90% of the mass of the sample. WPM: without physical modification. **Table S3.** Particle size distribution (mean ± standard deviation) of ground and original biochars used for testing 28-day compressive strength. D_10_, D_50_ and D_90_: the maximum diameter containing 10, 50 and 90% of the mass of the sample. WPM: without physical modification. **Figure S1.** The overall effect sizes of biochar addition on 7- and 28-day compressive strength of Portland cement composites. Each point represents effect sizes, and the size of the point represents the relative number of records compared to the total records. Grey bars represent 95CI. The vertical dash line represents the value of 0. The numbers of records are indicated in the brackets. **Figure S2.** Linear relationship between effect sizes of biochar addition on 7- and 28-day compressive strength of Portland cement composites. Points in the figure represent paired records. The simple linear regression line with 95% confidential intervals is shown, with the number of records (n) presented. The horizontal dash lines represent the value of 0.

## Data Availability

The datasets used or analyzed during the current study are available from the corresponding author upon reasonable request.
