# Supplementary material for: Biochar affects compressive strength of Portland cement composites: a meta-analysis
Source: Biochar. 2024 Mar 6;6(1):21. doi: 10.1007/s42773-024-00309-2 (PMC10917841; doi:10.1007/s42773-024-00309-2)
Supplement: Supplementary file 1 — Additional file 1: Table S1. List of papers used for meta-analysis with the parameters of 7- and 28-day compressive strength. Table S2. Particle size distribution (mean ± standard deviation) of ground and original biochars used for testing 7-day compressive strength. D10, D50 and D90: the maximum diameter containing 10%, 50% and 90% of the mass of the sample. WPM: without physical modification. Table S3. Particle size distribution (mean ± standard deviation) of ground and original biochars used for testing 28-day compressive strength. D10, D50 and D90: the maximum diameter containing 10, 50 and 90% of the mass of the sample. WPM: without physical modification. Figure S1. The overall effect sizes of biochar addition on 7- and 28-day compressive strength of Portland cement composites. Each point represents effect sizes, and the size of the point represents the relative number of records compared to the total records. Grey bars represent 95CI. The vertical dash line represents the value of 0. The numbers of records are indicated in the brackets. Figure S2. Linear relationship between effect sizes of biochar addition on 7- and 28-day compressive strength of Portland cement composites. Points in the figure represent paired records. The simple linear regression line with 95% confidential intervals is shown, with the number of records (n) presented. The horizontal dash lines represent the value of 0. [file 42773_2024_309_MOESM1_ESM.docx]

Supplementary information for

**Biochar affects compressive strength of Portland cement composites: a meta-analysis**

Zhihao Zhao^a^, Ali El-Naggar^a,^ ^b, c^, Johnson Kau^d^, Chris Olson^e^, Douglas Tomlinson^d^, Scott X. Chang^a *^

^a^ Department of Renewable Resources, 442 Earth Sciences Building, University of Alberta, Edmonton, Alberta T6G 2E3, Canada

^b^ Department of Soil Sciences, Faculty of Agriculture, Ain Shams University, Cairo 11241, Egypt

^c^ State Key Laboratory of Subtropical Silviculture, Zhejiang A&F University, Hangzhou, Zhejiang 311300, China

^d^ Department of Civil Engineering, 6-255 Donadeo Innovation Centre For Engineering, University of Alberta, Edmonton, Alberta T6G 2H5, Canada

^e^ Innovative Reduction Strategies Inc, PO Box 71022 Northtown PO, Edmonton, Alberta T5E 6J8, Canada

* Corresponding author. Email address: sxchang@ualberta.ca

Table S1. List of papers used for meta-analysis with the parameters of 7- and 28-day compressive strength.

| **No.** | **Papers included in the meta-analysis** | **7-day compressive strength** | **28-day compressive strength** |
| --- | --- | --- | --- |
| 1 | Ahmad, M.R., Chen, B., Duan, H., 2020. Improvement effect of pyrolyzed agro-food biochar on the properties of magnesium phosphate cement. Sci. Total Environ. 718, 137422. |  | ✓* |
| 2 | Akhtar, A., Sarmah, A.K., 2018. Novel biochar-concrete composites: manufacturing, characterization and evaluation of the mechanical properties. Sci. Total Environ. 616–617, 408–416. | ✓ | ✓ |
| 3 | Aziz, M.A., Zubair, M., Saleem, M., Alharthi, Y., Ashraf, N., Alotaibi, K.S., Aga, O., Al Eld, A.A.A., 2023. Mechanical, non-destructive, and thermal characterization of biochar-based mortar composite. Biomass Conversion and Biorefinery. | ✓ | ✓ |
| 4 | Belaadi, A., Boumaaza, M., Alshahrani, H., Bourchak, M., 2023. Optimization of Palm Rachis Biochar Waste Content and Temperature Effects on Predicting Bio-Mortar : ANN and RSM Modelling. Journal of Natural Fibers, 20(1), 2151547. | ✓ | ✓ |
| 5 | Chen, X., Li, J., Xue, Q., Huang, X., Liu, L., Poon, C.S., 2020. Sludge biochar as a green additive in cement-based composites: mechanical properties and hydration kinetics. Constr. Build. Mater. 262, 120723. | ✓ | ✓ |
| 6 | Chen, T., Zhao, L., Gao, X., Li, L., Qin, L., 2022. Modification of carbonation-cured cement mortar using biochar and its environmental evaluation. Cem. Concr. Compos. 134, 104764. | ✓ | ✓ |
| 7 | Chen, Z., Wu, N., Song, Y., Xiang, J., 2022. Modification of iron-tailings concrete with biochar and basalt fiber for sustainability. Sustainability 14, 10041. | ✓ | ✓ |
| 8 | Chen, L., Wang, L., Zhang, Y., Ruan, S., Mechtcherine, V., Tsang, D.C.W., 2022. Roles of biochar in cement-based stabilization/solidification of municipal solid waste incineration fly ash. Chem. Eng. J. 430, 132972. | ✓ | ✓ |
| 9 | De Carvalho Gomes, S., Zhou, J.L., Zeng, X., Long, G., 2022. Water treatment sludge conversion to biochar as cementitious material in cement composite. J. Environ. Manage. 306, 114463. | ✓ | ✓ |
| 10 | Gupta, S., 2021. Carbon sequestration in cementitious matrix containing pyrogenic carbon from waste biomass: a comparison of external and internal carbonation approach. J. Build. Eng. 43, 102910. | ✓ | ✓ |
| 11 | Gupta, S., Kua, H.W., Tan Cynthia, S.Y., 2017. Use of biochar-coated polypropylene fibers for carbon sequestration and physical improvement of mortar. Cem. Concr. Compos. 83, 171–187. | ✓ | ✓ |
| 12 | Gupta, S., Kua, H.W., Koh, H.J., 2018. Application of biochar from food and wood waste as green admixture for cement mortar. Sci. Total Environ. 619–620, 419–435. | ✓ | ✓ |
| 13 | Gupta, S., Kua, H.W., Pang, S.D., 2018. Biochar-mortar composite: manufacturing, evaluation of physical properties and economic viability. Constr. Build. Mater. 167, 874–889. | ✓ | ✓ |
| 14 | Gupta, S., Kua, H.W., 2018. Effect of water entrainment by pre-soaked biochar particles on strength and permeability of cement mortar. Constr. Build. Mater. 159, 107–125. | ✓ | ✓ |
| 15 | Gupta, S., Kua, H.W., Low, C.Y., 2018. Use of biochar as carbon sequestering additive in cement mortar. Cem. Concr. Compos. 87, 110–129. | ✓ | ✓ |
| 16 | Gupta, S., Kua, H.W., 2019. Carbonaceous micro-filler for cement: effect of particle size and dosage of biochar on fresh and hardened properties of cement mortar. Sci. Total Environ. 662, 952–962. |  | ✓ |
| 17 | Gupta S., Kua, H.W., Pang S.D., 2019. Biochar-concrete composite: manufacturing, characterization and performance evaluation at elevated temperature. Academic Journal of Civil Engineering, 37(2), 507-513. | ✓ | ✓ |
| 18 | Gupta, S., Krishnan, P., Kashani, A., Kua, H.W., 2020. Application of biochar from coconut and wood waste to reduce shrinkage and improve physical properties of silica fume-cement mortar. Constr. Build. Mater. 262, 120688. | ✓ | ✓ |
| 19 | Gupta, S., Palansooriya, K.N., Dissanayake, P.D., Ok, Y.S., Kua, H.W., 2020. Carbonaceous inserts from lignocellulosic and non-lignocellulosic sources in cement mortar: preparation conditions and its effect on hydration kinetics and physical properties. Constr. Build. Mater. 264, 120214. | ✓ | ✓ |
| 20 | Gupta, S., Kua, H.W., 2020. Combination of biochar and silica fume as partial cement replacement in mortar: performance evaluation under normal and elevated temperature. Waste Biomass Valorization 11, 2807–2824. | ✓ | ✓ |
| 21 | Gupta, S., Kua, H.W., Pang, S.D., 2020. Effect of biochar on mechanical and permeability properties of concrete exposed to elevated temperature. Constr. Build. Mater. 234, 117338. | ✓ | ✓ |
| 22 | Gupta, S., Kashani, A., Mahmood, A.H., Han, T., 2021. Carbon sequestration in cementitious composites using biochar and fly ash – effect on mechanical and durability properties. Constr. Build. Mater. 291, 123363. | ✓ | ✓ |
| 23 | Gupta, S., Muthukrishnan, S., Kua, H.W., 2021. Comparing influence of inert biochar and silica rich biochar on cement mortar – hydration kinetics and durability under chloride and sulfate environment. Constr. Build. Mater. 268, 121142. | ✓ |  |
| 24 | Gupta, S., Kashani, A., 2021. Utilization of biochar from unwashed peanut shell in cementitious building materials – effect on early age properties and environmental benefits. Fuel Process. Technol. 218, 106841. | ✓ |  |
| 25 | Gupta, S., Mahmood, A.H., 2022. A multi-method investigation into rheological properties, hydration, and early-age strength of cement composites with admixtures recovered from inorganic and bio-based waste streams. Constr. Build. Mater. 347, 128529. | ✓ | ✓ |
| 26 | Gupta, S., Mahmood, A.H., 2022. A multi-method investigation into rheological properties, hydration, and early-age strength of cement composites with admixtures recovered from inorganic and bio-based waste streams. Constr. Build. Mater. 347, 128529. | ✓ |  |
| 27 | Haque, M.I., Khan, R.I., Ashraf, W., Pendse, H., 2021. Production of sustainable, low-permeable and self-sensing cementitious composites using biochar. Sustain. Mater. Technol. 28, e00279. | ✓ | ✓ |
| 28 | Haris Javed, M., Ali Sikandar, M., Ahmad, W., Tariq Bashir, M., Alrowais, R., Bilal Wadud, M., 2022. Effect of various biochars on physical, mechanical, and microstructural characteristics of cement pastes and mortars. J. Build. Eng. 57, 104850. |  | ✓ |
| 39 | Jafari, A., Sadeghian, P., 2023. Influence of biochar and recycled gypsum on the strength and microstructure of conventional and sustainable cementitious composites. Construction and Building Materials, 408, 133715. | ✓ | ✓ |
| 30 | Khalid, A., Khushnood, R.A., Mahmood, A., 2019. Impact of pyrolytic carbonaceous nano inerts addition on fracture and electromagnetic interference shielding characteristics of cementitious composites. Theor. Appl. Fract. Mech. 103, 102320. |  | ✓ |
| 31 | Khan, K., Aziz, M.A., Zubair, M., Amin, M.N., 2022. Biochar produced from Saudi agriculture waste as a cement additive for improved mechanical and durability properties—SWOT analysis and techno-economic assessment. Materials 15, 5345. | ✓ | ✓ |
| 32 | Kim, Y.U., Park, J.H., Yun, B.Y., Yang, S., Wi, S., Kim, S., 2021. Mechanical and thermal properties of artificial stone finishing materials mixed with PCM impregnated lightweight aggregate and carbon material. Constr. Build. Mater. 272, 121882. |  | ✓ |
| 33 | Liu, W., Li, K., Xu, S., 2022. Utilizing bamboo biochar in cement mortar as a bio-modifier to improve the compressive strength and crack-resistance fracture ability. Constr. Build. Mater. 327, 126917. | ✓ | ✓ |
| 34 | Li, Z., Xue, W., Zhou, W., 2023. Mechanical Properties of Concrete with Different Carya Cathayensis Peel Biochar Additions. Sustainability, 15(6), 4874. |  | ✓ |
| 35 | Maljaee, H., Paiva, H., Madadi, R., Tarelho, L.A.C., Morais, M., Ferreira, V.M., 2021. Effect of cement partial substitution by waste-based biochar in mortars properties. Constr. Build. Mater. 301, 124074. | ✓ | ✓ |
| 36 | Mo, L., Fang, J., Huang, B., Wang, A., Deng, M., 2019. Combined effects of biochar and MgO expansive additive on the autogenous shrinkage, internal relative humidity and compressive strength of cement pastes. Constr. Build. Mater. 229, 116877. | ✓ | ✓ |
| 37 | Navaratnam, S., Wijaya, H., Rajeev, P., Mendis, P., Nguyen, K., 2021. Residual stress-strain relationship for the biochar-based mortar after exposure to elevated temperature. Case Stud. Constr. Mater. 14, e00540. |  | ✓ |
| 38 | Park, J.H., Kim, Y.U., Jeon, J., Yun, B.Y., Kang, Y., Kim, S., 2021. Analysis of biochar-mortar composite as a humidity control material to improve the building energy and hygrothermal performance. Sci. Total Environ. 775, 145552. | ✓ | ✓ |
| 39 | Qin, Y., Pang, X., Tan, K., Bao, T., 2021. Evaluation of pervious concrete performance with pulverized biochar as cement replacement. Cem. Concr. Compos. 119, 104022. | ✓ | ✓ |
| 40 | Qing, L., Zhang, H., Zhang, Z., 2023. Effect of biochar on compressive strength and fracture performance of concrete. Journal of Building Engineering, 78, 107587. | ✓ | ✓ |
| 41 | Restuccia, L., Ferro, G.A., 2016. Promising low cost carbon-based materials to improve strength and toughness in cement composites. Constr. Build. Mater. 126, 1034–1043. | ✓ | ✓ |
| 42 | Restuccia, L., Ferro, G.A., 2018. Influence of filler size on the mechanical properties of cement-based composites. Fatigue Fract. Eng. Mater. Struct. 41, 797–805. | ✓ | ✓ |
| 43 | Roychand, R., Patel, S., Halder, P., Kundu, S., Hampton, J., Bergmann, D., Surapaneni, A., Shah, K., Pramanik, B.K., 2021. Recycling biosolids as cement composites in raw, pyrolyzed and ashed forms: a waste utilisation approach to support circular economy. J. Build. Eng. 38, 102199. | ✓ | ✓ |
| 44 | Sirico, A., Belletti, B., Bernardi, P., Malcevschi, A., Pagliari, F., Fornoni, P., Moretti, E., 2022. Effects of biochar addition on long-term behavior of concrete. Theoretical and Applied Fracture Mechanics, 122, 103626. | ✓ | ✓ |
| 45 | Suarez-Riera, D., Lavagna, L., Bartoli, M., Giorcelli, M., Pavese, M., Tagliaferro, A., 2022. The influence of biochar shape on cement-based materials. Mag. Concr. Res. 1–6. |  | ✓ |
| 46 | Tan, K., Pang, X., Qin, Y., Wang, J., 2020. Properties of cement mortar containing pulverized biochar pyrolyzed at different temperatures. Constr. Build. Mater. 263, 120616. | ✓ | ✓ |
| 47 | Tan, K., Qin, Y., Wang, J., 2022. Evaluation of the properties and carbon sequestration potential of biochar-modified pervious concrete. Constr. Build. Mater. 314, 125648. | ✓ | ✓ |
| 48 | Wang, L., Chen, L., Tsang, D.C.W., Kua, H.W., Yang, J., Ok, Y.S., Ding, S., Hou, D., Poon, C.S., 2019. The roles of biochar as green admixture for sediment-based construction products. Cem. Concr. Compos. 104, 103348. | ✓ | ✓ |
| 49 | Yang, X., Wang, X.-Y., 2021. Hydration-strength-durability-workability of biochar-cement binary blends. J. Build. Eng. 42, 103064. | ✓ | ✓ |
| 50 | Zhang, Y., Xu, H., Fang, S., Li, D., Xue, W., Chen, B., Zhao, L., 2022. Biochar as additive for improved building performances and heavy metals solidification of sediment-based lightweight concrete. Environ. Sci. Pollut. Res. 30, 4137–4150. |  | ✓ |
| 51 | Zhang, Y., Maierdan, Y., Guo, T., Chen, B., Fang, S., Zhao, L., 2022. Biochar as carbon sequestration material combines with sewage sludge incineration ash to prepare lightweight concrete. Constr. Build. Mater. 343, 128116. |  | ✓ |

*: Indicating that a particular publication includes data for the parameter listed in the column heading.

Table S2. Particle size distribution (mean ± standard deviation) of ground and original biochars used for testing 7-day compressive strength. D_10_, D_50_ and D_90_: the maximum diameter containing 10%, 50% and 90% of the mass of the sample. WPM: without physical modification.

| **Modification** | **Cement** |  |  |  | **Biochar** |  |  | **Sample size (n)** |
| --- | --- | --- | --- | --- | --- | --- | --- | --- |
|  | **D_10_ (µm)** | **D_50_ (µm)** | **D_90_ (µm)** |  | **D_10_ (µm)** | **D_50_ (µm)** | **D_90_ (µm)** |  |
| Grinding | 3.6 ± 1.6 | 17.1 ± 5.1 | 39.3 ± 8.4 |  | 3.4 ± 1.4 | 13.4 ± 9.1 | 46.4 ± 36.1 | 63 |
| WPM | 2.4 ± 0.9 | 14.9 ± 3.1 | 45.1 ± 5.0 |  | 4.2 ± 3.1 | 63.6 ± 65.4 | 280.0 ± 293 | 54 |

Table S3. Particle size distribution (mean ± standard deviation) of ground and original biochars used for testing 28-day compressive strength. D_10_, D_50_ and D_90_: the maximum diameter containing 10, 50 and 90% of the mass of the sample. WPM: without physical modification.

| **Modification** | **Cement** |  |  |  | **Biochar** |  |  | **Sample size (n)** |
| --- | --- | --- | --- | --- | --- | --- | --- | --- |
|  | **D_10_ (µm)** | **D_50_ (µm)** | **D_90_ (µm)** |  | **D_10_ (µm)** | **D_50_ (µm)** | **D_90_ (µm)** |  |
| Grinding | 3.4 ± 1.5 | 16.3 ± 4.4 | 41.4 ± 11.2 |  | 3.2 ± 1.1 | 11.8 ± 3.9 | 43.6 ± 22.5 | 65 |
| WPM | 3.1 ± 1.3 | 15.0 ± 2.4 | 43.5 ± 4.5 |  | 3.3 ± 2.9 | 45.2 ± 61.0 | 201.0 ± 271 | 78 |

Figure S1. The overall effect sizes of biochar addition on 7- and 28-day compressive strength of Portland cement composites. Each point represents effect sizes, and the size of the point represents the relative number of records compared to the total records. Grey bars represent 95CI. The vertical dash line represents the value of 0. The numbers of records are indicated in the brackets.

Figure S2. Linear relationship between effect sizes of biochar addition on 7- and 28-day compressive strength of Portland cement composites. Points in the figure represent paired records. The simple linear regression line with 95% confidential intervals is shown, with the number of records (n) presented. The horizontal dash lines represent the value of 0.
